# Strain Differences in Light-Induced Retinopathy

**DOI:** 10.1371/journal.pone.0158082

**Published:** 2016-06-29

**Authors:** Anna Polosa, Hyba Bessaklia, Pierre Lachapelle

**Affiliations:** 1 Department of Neurology and Neurosurgery, McGill University, Montreal, Quebec, Canada; 2 Department of Ophthalmology, Research Institute of the McGill University Health Centre, Montreal, Quebec, Canada; Dalhousie University, CANADA

## Abstract

The purpose of this study was to better understand the role of ocular pigmentation and genetics in light-induced retinal damage. Adult pigmented [Long Evans (LE) and Brown Norway (BN)] and albino [Sprague Dawley (SD) and Lewis (LW)] rats were exposed to a bright cyclic light for 6 consecutive days and where compared with juvenile animals exposed to the same bright light environment from postnatal age 14 to 28. Flash ERGs and retinal histology were performed at predetermined days (D) post-light exposure. At D1, ERGs were similar in all adult groups with no recordable a-waves and residual b-waves. A transient recovery was noticed at D30 in the LW and LE only [b-wave: 18% and 25% of their original amplitude respectively]. Histology revealed that BN retina was the most damaged, while LE retina was best preserved. SD and LW rats were almost as damaged as BN rats. In contrast, the retina of juvenile BN was almost as resistant to the bright light exposure as that of juvenile LE rats. Our results strongly suggest that, although ocular pigmentation and genetic background are important factors in regulating the severity of light-induced retinal damage, the age of the animal at the onset of light exposure appears to be the most important determining factor.

## Introduction

In animal models of light damage, when strain differences are taken into consideration, the retina of pigmented animals is usually reported to be more resistant to damage compared to that of albino strains [1–3], suggesting that ocular pigmentation does indeed protect the retina from light-induced damages. Of interest, it was also shown that two phenotypically (i.e. albino mice) identical strains of mice but with different genetic backgrounds (the albino BALB/cByJ and the albino C57BL/6J-c^2J^ mice) demonstrated strikingly different susceptibility to light damage [4]; the former showing a near complete destruction of the outer retina while the latter was minimally affected. The authors concluded that, apart from pigmentation, genetic factors, must also be taken into consideration when performing strain-related studies on light-induced retinal damage.

Given that some strains of albino animals appear to be relatively better equipped than others to deal with a light-induced oxidative stress, is it also possible that some strains of pigmented animals do poorly when subjected to the same oxidative stress? In other words, is it fair to say that pigmented strains are always better off when exposed to a bright luminous environment? Although this was not the objective, this claim was recently put to test in a study which compared the protective effect of blueberry extract (potent antioxidant) on the retina of pigmented Brown-Norway (BN) and albino Wistar (WS) rats exposed to bright light [5]. This study not only showed that the retina of adult pigmented BN rats was more susceptible to bright light exposure compared to the retina of albino WS rats, it was also shown to be less responsive to the antioxidant therapy [5].

Consequently, in order to better appreciate the contribution of ocular pigmentation and genetics in protecting the retina from light-induced oxidative damage, we compared inbred (Brown Norway and Lewis rats) and outbred (Sprague-Dawley and Long-Evans) albino (Lewis and Sprague-Dawley) and pigmented (Brown Norway and Long-Evans) rat stains in their response to bright light exposure. Our results revealed that the retina of BN rats was indeed highly susceptible to bright-light damage, almost to the same extent as albino strains and significantly more than the LE rats. However this high vulnerability to light damage could only be demonstrated in adult animals since the retina of juvenile BN rats, like that of other strains of juvenile rats [6–10] was resistant to light exposure. Our results show that although both ocular pigmentation and the genetic background of the animal are key factors that determine retinal susceptibility to light-induced damage, the age of the animal at the onset of light exposure appears to be the most important factor in predicting the severity of the ensuing light induced retinopathy.

## Methods

All experiments were performed in compliance with the ARVO Statement for the Use of Animals in Ophthalmic and Vision Research and were approved by the McGill University-Montreal Children's Hospital Animal Care Committee. Animals were anesthetized with a cocktail of ketamine-xylazine and euthanized with CO_2_.

### Animals

Four different strains of adult rats were used in this study: two pigmented [Brown Norway (BN) and Long Evans (LE) rats] and two albino [Sprague Dawley (SD) and Lewis (LW) rats]. All animals were purchased from Charles River Laboratories (St-Constant, Qc, Canada) and were housed in a cyclic 12-hour light/dark light environment of 80 lux for two weeks prior to the light exposure in order to remove the previously reported “light history” effect [11]. All the animals were aged 60 days old (P60) at the onset of the light exposure. A total of 12 animals per strain was used.

One litter of BN and one of LE rats (n = 6 pups per strain; Charles River Laboratories, St-Constant, Qc, Canada) were also used to evaluate if age at onset of the light exposure could explain the significant difference we noted in the retinal susceptibility to light damage between the two pigmented strains. The rat pups were aged P10 upon arrival and were placed, prior to the light exposure, in the same standard luminous environment of the animal care facility as that used for the adult rats.

### Bright light exposure

Adult rats (maximum three per cage) were placed in transparent Plexiglas^™^ cages and exposed to a bright luminous environment of 10 000 lux (12h light/12 hours dark) for a total of 6 consecutive days, following a protocol previously described by us [6, 12]. As previously suggested [10,13], the pupils of pigmented strains were maintained dilated throughout the light exposure regimen (P60-P66) with drops of atropine sulphate 1.0% (Chauvin Pharmaceuticals Ltd). Pupil diameter was monitored daily and drops were added when necessary. Age-matched controls (n = 3 to 4 per strains) were housed under the normal lighting conditions of the animal care facility.

Juvenile animals were exposed to the same light intensity as the adult groups, but for a total of 14 days (12h light/12h dark), following a protocol previously described by us [6, 12]. The light exposure started at postnatal day (P) 14 and ended at P28. Pupil dilation was also maintained throughout the light exposure period.

### Evaluation of the retinal function

Functional assessment of retinal damage was performed with the use of flash electroretinograms (fERGs) following a protocol previously described by us [6,12]. For the adult groups, three different time points, post-bright light exposure, were chosen, namely: [day (D) 1, 15 and 31]. Animals that still showed signs of retinal function at D31 were re-assessed at long term [between 1.0 to 1.5 years post-light exposure]. Similarly, juvenile BN and LE rats were tested at P30 (immediate effects of light exposure) and at P60 (30 days following the cessation of light exposure).

Analysis of fERG responses [Prism 6.0 software; Graph Pad, San Diego, CA)] included amplitude measurements of the a-waves and b-waves, as reported previously [6,12]. The rodVmax was determined based on the threshold of appearance of the a-wave which was defined as a negative deflection of an amplitude double of that of the noise level (as previously published: [14]). The intensity at which rodVmax amplitudes were calculated was also reported in all four strains (control and exposed).

### Evaluation of the retinal structure

Retinal histology was performed as previously reported by us [12]. Briefly, the left eyes (n = 3 per group) were fixed either with 4% paraformaldehyde or 4% glutaraldehyde. Retinal sections (1.0 μm-thick) were then bisected along the vertical meridian (superior-inferior axis) of the eye passing through the optic nerve head (ONH), collected on glass slides and stained with 0.1% toluidine blue. Retinal images and thickness measurements were performed with the AxioVision 4.8 software (Carl Zeiss Canada Ltd, CA). Analysis of retinal samples included: 1-a retinal reconstruction of the superior and inferior retinas [composed of 12 to 14 (segments S1 to S12 or S14) connecting histological segments of 75μm in width, each sectioned at every 340μm from the ONH to the ora serrata] and 2- the thickness of the outer nuclear layer (ONL) at each of the 12–14 segments (spidergraph representation).

### Melanin pigment distribution

For each of the 12–14 retinal segments described above, the density of melanin pigments was determined using a subjective scale ranging from grade 1 to 4. Grade 1 identified retinal segments were the melanin pigment was absent; Grade 2, a discontinuous one to two pigment thick layer of melanin; Grade 3, a continuous one to three pigment thick layer of melanin; and Grade 4, a densely packed layer of melanin pigments (> than 3 pigments thick continuous layer). In order to better visualize the distribution of melanin pigments, the colour images were changed to black and white. Furthermore, the images were trimmed down to accentuate the layer of melanin pigments. Arrows were also added to identify the melanin pigment layer and regions lacking melanin pigments were underlined in red.

### Data analysis

Statistical significance was determined using either a Student’s *t*-test or a one-way factor ANOVA followed by the Tukey post hoc test (Prism 6.0 software; Graph Pad, San Diego, CA). For all statistical tests performed, a *p* value < 0.05 was considered as statistically significant. All values are reported as mean ± 1 standard deviation (SD).

## Results

### Retinal function in adult LIR

**Fig 1:**
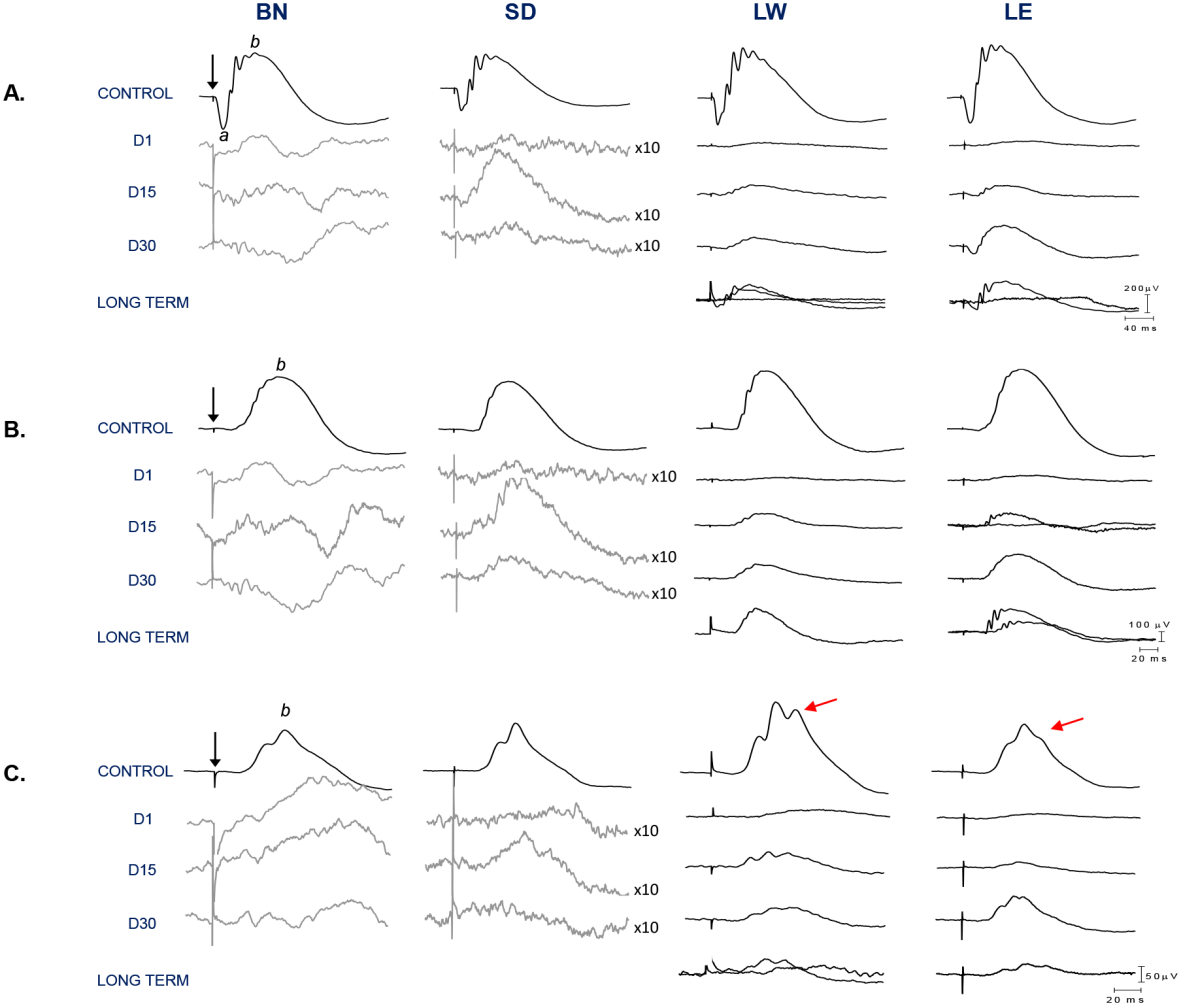
Representative scotopic [rod-cone responses (A) and rodVmax (B)] and photopic (C) ERGs recorded from four different control and light-exposed rat strains (BN, SD, LW, LE) 1, 15 and 31 days following the end of the bright light exposure as well as at long term (LW and LE only). In order to better appreciate residual responses, some tracings were amplified 10 times (gray waveforms). In addition, in LW and LE rats (at D15 and at long term), individual waveforms from each animal are presented separately to highlight variations in amplitude within the same group. Abbreviations: Brown Norway (BN), Sprague-Dawley (SD), Lewis (LW), Long Evans (LE), a-wave (a), b-wave (b) and days (D). Calibration: horizontal: rod-cone response: 200μV, rod Vmax: 100 μV and photopic response: 50 μV, for black waveforms; vertical: 40ms. A 20ms stimulus baseline is included in all tracings. Vertical arrows indicate the flash onset.

**Fig 2:**
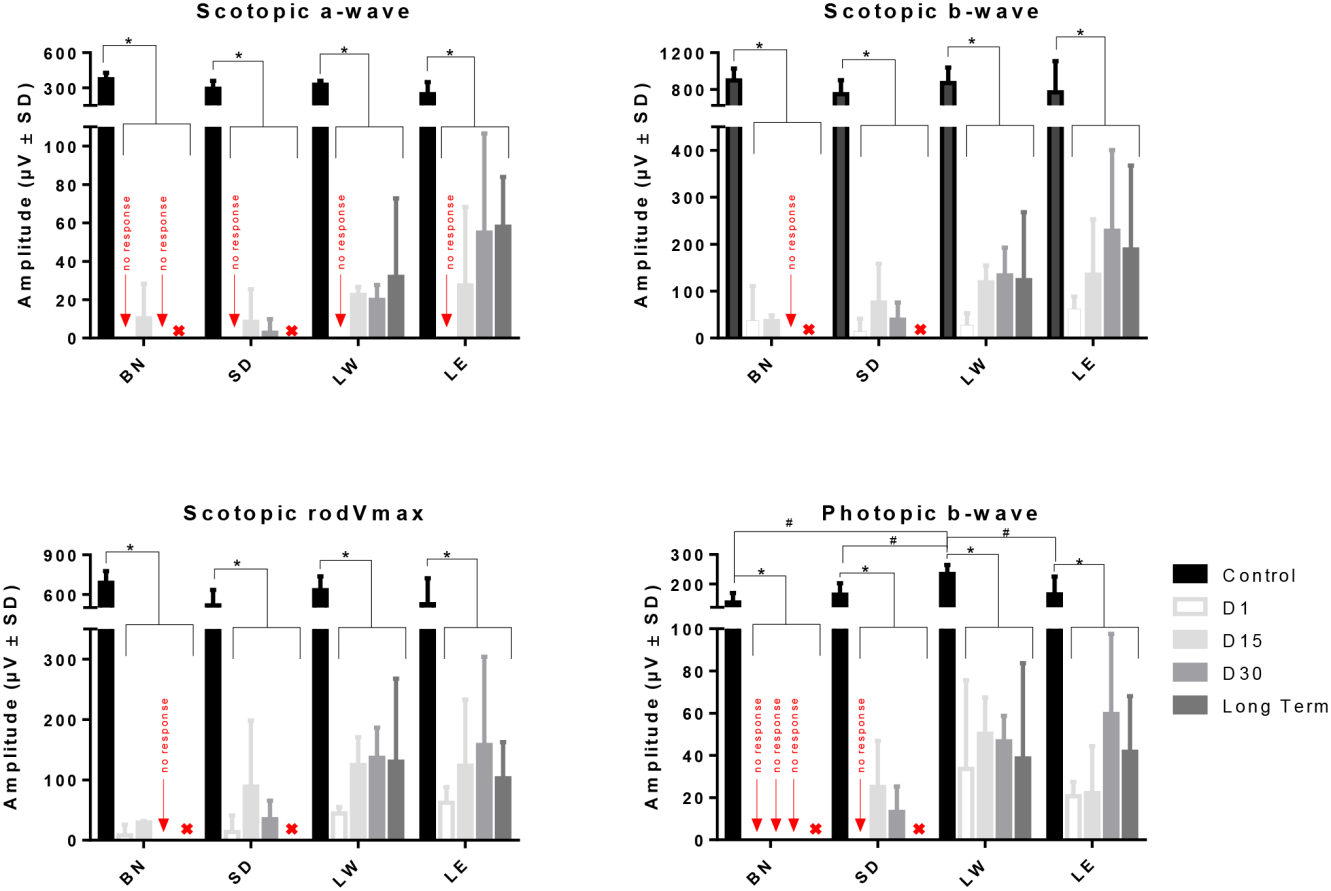
Graphic representation of the global retinal function (scotopic a-wave, b-wave and rodVmax and photopic b-wave) in four different strains of rats (BN, SD, LW and LE) at different time points following the cessation of the bright light insult. Asterisks represent statistically significant differences (p<0.05) between light-exposed and aged-matched control groups, while the symbol # represent statistically significant differences between LW and other strains. Amplitudes are reported as mean 1±SD. Abbreviations: Brown Norway (BN), Sprague-Dawley (SD), Lewis (LW) and Long Evans (LE).

Representative scotopic rod-cone (A), rodVmax (B) and photopic (C) ERGs obtained from control and light exposed rats (BN, SD, LW, LE) are illustrated in Fig 1. As exemplified with the waveforms shown at Fig 1 as well as with the group data of Fig 2, control scotopic ERGs were of similar amplitude (Fig 2; p>0.05) and morphologies (Fig 1) in all four strains. In contrast, although the morphologies of the photopic ERGs (Fig 1) were similar (albeit an extra oscillatory on the b-wave descent of LW and LE’s ERGs), significantly higher responses were obtained from LW rats compared to the other strains [photopic b-wave amplitude: 145.81±20.59μV, 163.114±38.81, 234.19±30.31 and 171.75±57.51 for BN, SD, LW and LE, respectively, p<0.05]. No significant (p>0.05) difference was found between the other three strains. One day following light exposure, only residual (non-reproducible) scotopic ERGs (no a-wave and residual b-waves) were recorded from all four strains, while cone responses were obtained only from LW and LE rats (Figs 1 and 2). These responses were of significantly reduced amplitudes compared to controls [14% and 12% of control (p<0.05), respectively (Fig 2)]. ERGs of recognisable morphologies could only be recorded from LW and LE rats (D15, D31 and long term recordings) while only severely depressed ERGs could be recorded from BN and SD rats irrespective of time post exposure.

Group data illustrated at Fig 2 reveals that a small recovery of retinal function was noted in all four strains. In BN and SD rats, this recovery peaked at D15 after which a decline in retinal function took place. By D31, only residual responses were recorded in BN rats (rod and cone functions), while SD, LW and LE rats had recovered 5% (mean of rod and cone ERG parameters), 18% and 25% of their original amplitude, respectively. Close analysis revealed that in both SD and LW strains, a similar degree of recovery was observed between scotopic and photopic responses [rodVmax: 6% and 22%; photopic b-wave: 8% and 20% of control, respectively]. In contrast, in LE rats, a greater recovery was observed for the cone function than for the rod function [rodVmax: 24%; photopic b-wave: 35% of control]. Statistical analysis showed that at D31, while no significant differences (p>0.05) were observed between LW and LE strains, the latter two strains were significantly different (p<0.05) from SD rats. With further aging (long term data), the retinal function remained similar between LW and LE strains (only those two strains were tested at long term) and to that measured at D31 (p>0.05) (Fig 2).

In respect to the sensitivity of the rod system, in control groups, no significant strain related differences (p>0.05) were observed in the light intensity needed to elicit rodVmax values [RodVmax intensity (in log.cd.m^-2^): -2.1±0.6 (BN), -2.2±0.5 (SD), -2.5±0.2 (LW) and -2.1±0.5 (LE)] (S1 Table). In contrast, a significant shift (p<0.05) in rodVmax intensity was observed in light exposed animals, except for LE rats (p>0.05) [RodVmax intensity at D1 (in log.cd.m^-2^): 0.9±0.0 (BN), 0.9±0.0 (SD), 0.9±0.0 (LW) and -1.1±0.3 (LE)]. At long term, only in LW rats was the intensity of rodVmax statistically significant (p<0.05) from controls.

### Retinal histology in adult LIR

**Fig 3:**
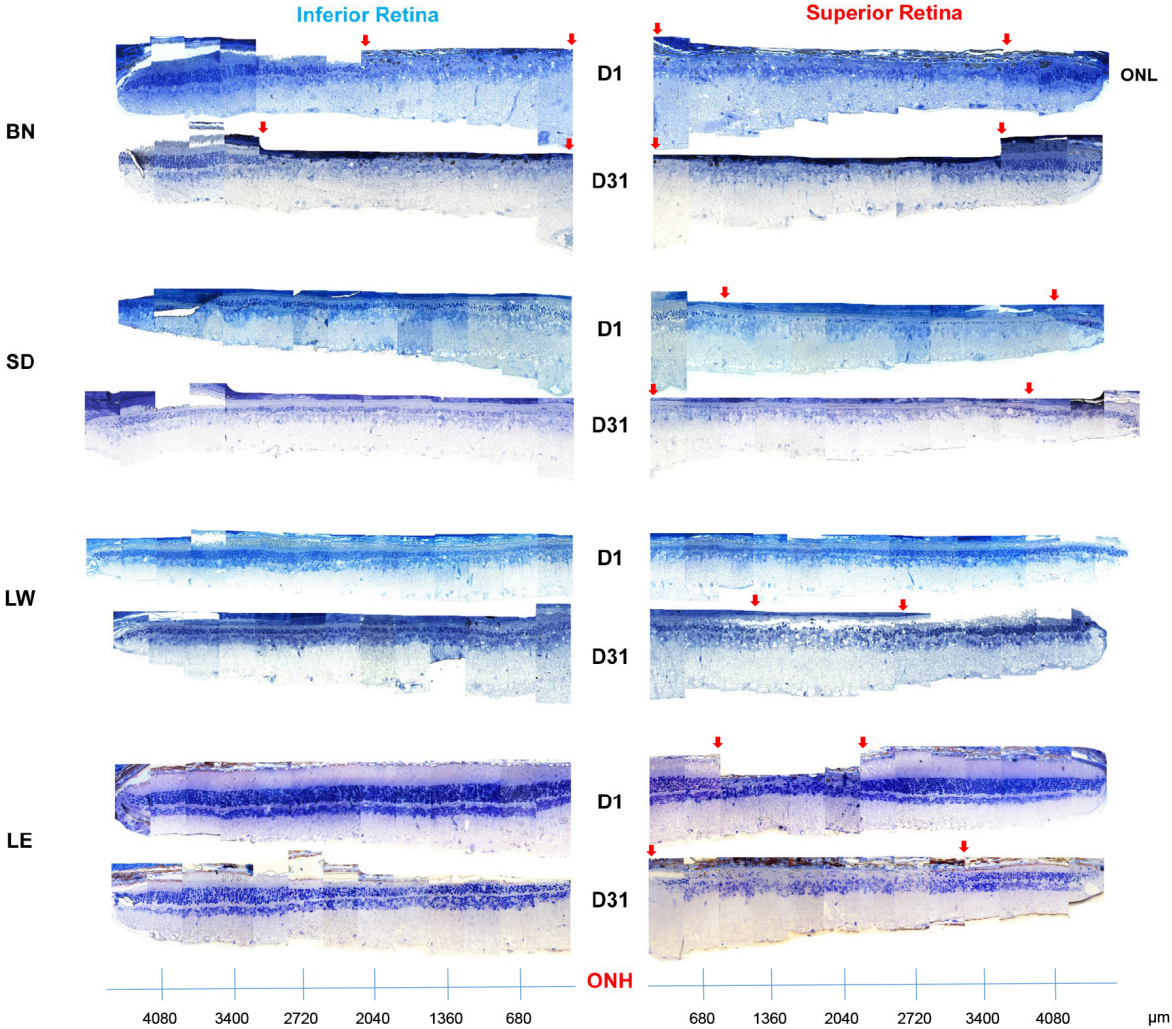
Representative reconstruction of the inferior (left) and superior (right) retina (composed of 12–13 consecutive histological segments of 75μm in width, each sectioned at every 340μm from the ONH to the ora serrate of each hemiretina) obtained from four different strains of adult light-exposed rats 1 day and 31 days following light exposure. Abbreviations: Optic nerve head (ONH), outer nuclear layer (ONL), Brown Norway (BN), Sprague-Dawley (SD), Lewis (LW) and Long Evans (LE). Red arrows indicate the portion of the retina devoid of photoreceptors.

**Fig 4:**
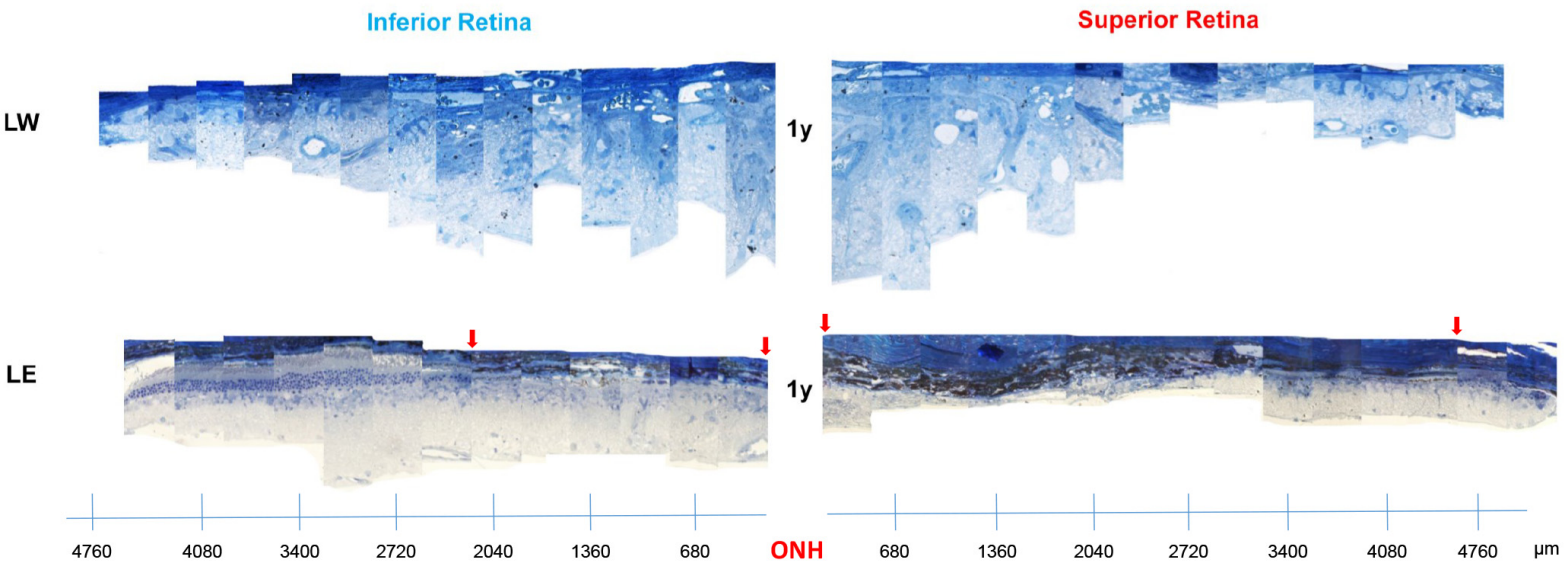
Representative reconstruction of the inferior (left) and superior (right) retina (composed of 12–13 consecutive histological segments of 75μm in width, each sectioned at every 340μm from the ONH to the ora serrate of each hemiretina) obtained from two different strains (LW and LE) of adult light-exposed rats over 1 year following light exposure. Abbreviations: Optic nerve head (ONH), Lewis (LW) and Long Evans (LE). Red arrows indicate the portion of the retina devoid of photoreceptors.

**Fig 5:**
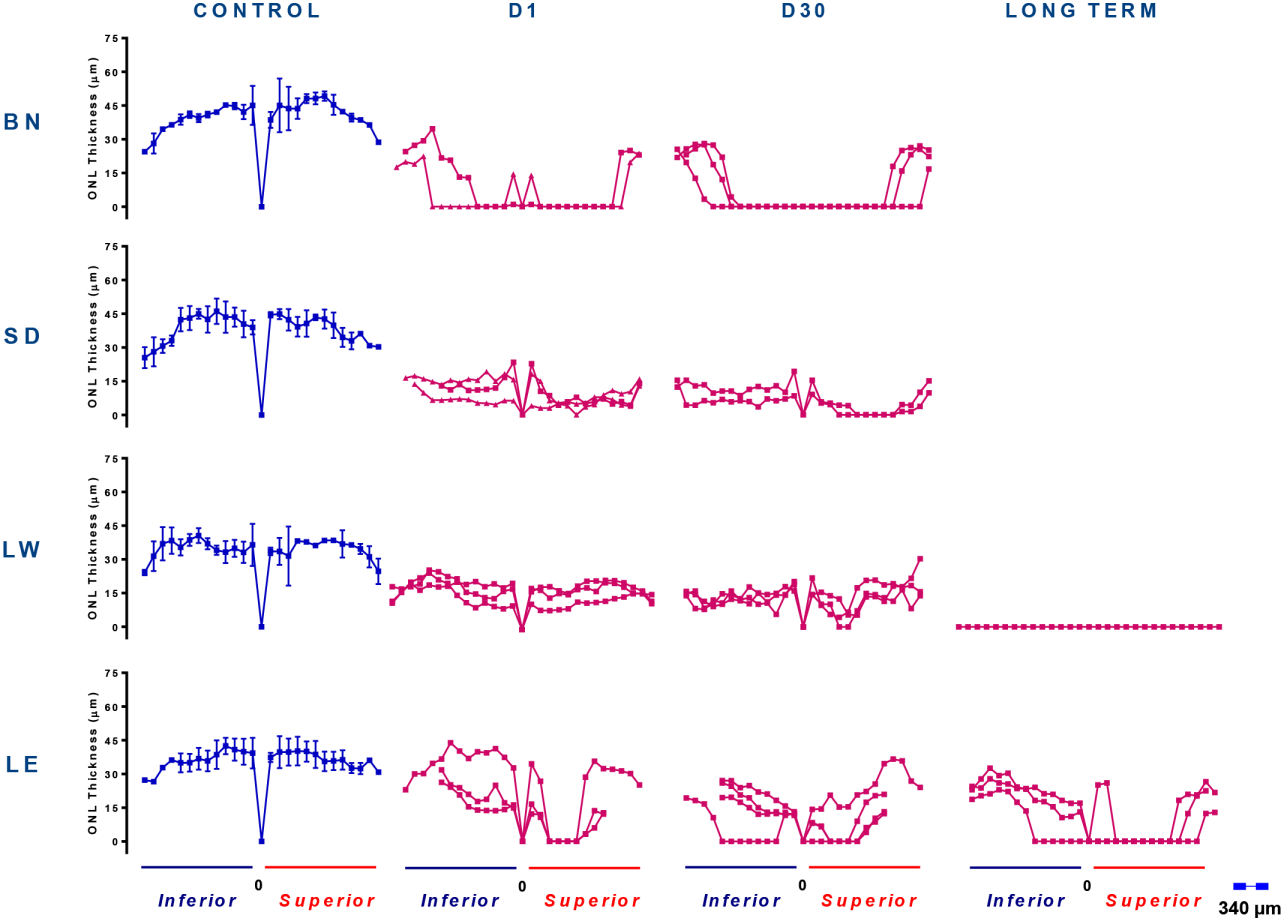
Spidergraph representation of ONL loss along the inferior (right) and superior (left) axis in control (blue) and light exposed (pink) rats shown for the four stains and at different time points following bright light exposure. Measurements were taken at every 340μm from the optic nerve head towards the ora serrate in both hemiretinas. Abbreviations: Optic nerve head (ONH), Brown Norway (BN), Sprague-Dawley (SD), Lewis (LW) and Long Evans (LE).

As illustrated at Figs 3–5, all four strains were significantly affected by the bright light exposure, albeit to different degrees. Measurements of outer nuclear layer (ONL) thickness revealed that BN rats were the most affected by the bright light exposure (Fig 5). One day following bright light exposure, the retina of BN rats was almost completely devoid of photoreceptors, except for the far periphery. As shown with the retinal reconstructions (Fig 3; an enlarged view of selected images is presented at S1 Fig) and spidergraphs of ONL thickness measurements (Fig 5), more photoreceptors survived at the periphery of the inferior retina compared to all of the superior retina [total width devoid of photoreceptors (as indicated with red arrows): from the optic nerve head (ONH) to 2020μm (inferior) and to 3800μm (superior)]. The SD rats were the second most affected strain. At D1, the retina of SD rats still presented with a single row of photoreceptors across most of the superior retina, while two to four rows were observed in the inferior retina (Figs 3 and 5). In comparison, damages to the retina of LW and LE rats were less severe. In the LW rats, the ONL was 2 to 3 nuclei thick centrally and 4 to 6 nuclei thick at the periphery (superior and inferior retinas). Finally, the strain that showed the highest resistance to light damage was the LE rat, where photoreceptor loss was mainly restricted to a small area of the superior retina (between 1000μm and 2700μm from the optic nerve head). No strain-related differences in ONL thickness were observed in the control groups (Fig 3).

With further aging (D31), retinal reconstructions (Fig 3) and spidergraphs of ONL thickness measurements (Fig 5) show that in BN rats, while the superior retina remained relatively stable (most of the superior retina was destroyed during the light exposure), the area devoid of photoreceptors in the inferior retina increased [total width devoid of photoreceptors (as indicated with red arrows): from the optic nerve head (ONH) to 3700μm (superior) and to 3400μm (inferior)]. In SD rats, the superior retina was now almost completely devoid of photoreceptors (only two to four nuclei rows remained at the periphery between 3400μm for the ONH and the ora serrata), while a two to four nuclei thick ONL layer was still observed across the inferior retina. In LW rats, while no hemiretinal differences were noted at D1, at D31, a small portion of the center of the superior retina was now lacking photoreceptors (between 1300μm and 2200μm from the optic nerve head). Finally, in LE rats, the region of the superior retina devoid of photoreceptors also increased in width, now covering an area between 600μm and 3300μm from the optic nerve head. An important thinning of ONL was also observed in the inferior retina, where the central part of the outer retina [three to five nuclei rows] was more reduced in thickness than the peripheral one [seven to nine nuclei rows] (Fig 5).

One year after the cessation of the bright light insult, the retinas of all three LW rats were completely devoid of photoreceptors, while the inner retina was significantly disorganized (inner retinal and choroid vessels invasion, retinal scaring, vacuolization, etc.) (Fig 4 and S2 Fig). In LE rats, most of the central part of the superior hemiretina (outer and/or inner layers) was almost completely destroyed and, where the damage was most pronounced, clumps of inner retinal cells could also be seen (Fig 4).

### Retinal function in juvenile LIR

**Fig 6:**
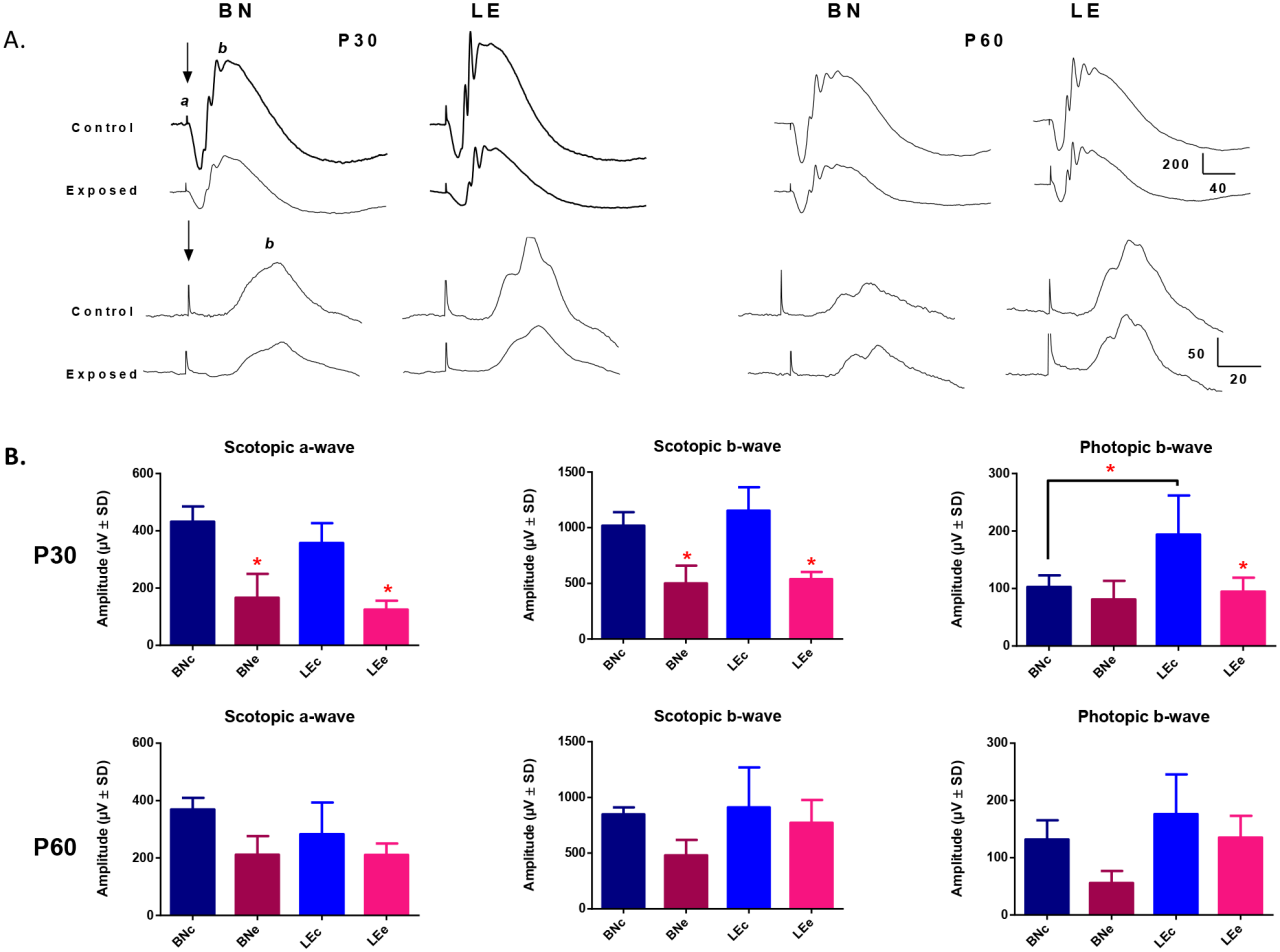
**(A)** Representative scotopic (rod-cone responses; top waveforms) and photopic (cone responses; bottom waveforms) ERGs recorded from juvenile BN and LE control and light-exposed rats at P30 and P60. Abbreviations: Brown Norway (BN), Long Evans (LE), a-wave (a) and b-wave (b). Calibration: horizontal: 200μV and 50μV; vertical: 40ms and 20ms, for scotopic and photopic waveforms respectively. A 20ms stimulus baseline is included in all tracings. Vertical arrows indicate the flash onset. **(B)** Graphic representation of the global retinal function (scotopic a-wave, b-wave and photopic b-wave) recorded juvenile BN and LE control and light-exposed rats at P30 and P60. Asterisks represent statistically significant differences (p<0.05) between light-exposed and aged-matched control groups. Amplitudes are reported as mean 1±SD. Abbreviations: Brown Norway (BN), Long Evans (LE), control (c) and exposed (e).

Given the highest vulnerability of the adult BN retina to light damage, we examined if this difference also characterised the retina of juvenile BN rats. Fig 6A compares scotopic and photopic ERGs recorded from control and light exposed juvenile BN and LE rats at age P30 and at P60. In control (unexposed) juvenile rats, both strains yielded scotopic responses (a- and b-waves) that were of similar amplitudes (p>0.05; Fig 6B) and morphologies (Fig 6A) at both time points (i.e. P30 and P60). In contrast, photopic responses were morphologically different (Fig 6A), the amplitude measured being significantly smaller in BN rats compared to LE rats [at P30: 102.93±19.98 vs 194.19±67.83μV (p<0.05); at P60: 145.81±20.59μV vs 176.02±69.30μV (p>0.05), BN and LE rats, respectively]. No significant age dependent differences were observed in both groups (p>0.05).

Bright light exposure significantly attenuated (and to the same extent) the scotopic responses of both strains [at P30 a-wave: 38% and 35%; b-wave: 49% and 47% of controls (p<0.05), for the BN and LE rats, respectively]. With time, a more pronounced recovery of function was measured in LE rats [at P60 a-wave: 57% and 74%; b-wave: 57% and 85% of respective controls, for the BN and LE rats, respectively (p<0.05)]. At P30, cone function was significantly reduced in the LE rats only [49% of control, p<0.05], but returned to normal values by P60 [77% of controls, p>0.05)]. Bright light exposure had no significant effect on the cone function of BN rats at P30 (p>0.05). However, by P60, the cone response in BN rats was lower (p>0.05) compared to values at P30. Furthermore, although no significant differences between the two strains were observed at P60, photopic ERG amplitudes measured for BN rats at P60 were also lower compared to those of LE rats at P60 (Fig 6).

### Retinal structure in juvenile LIR

**Fig 7:**
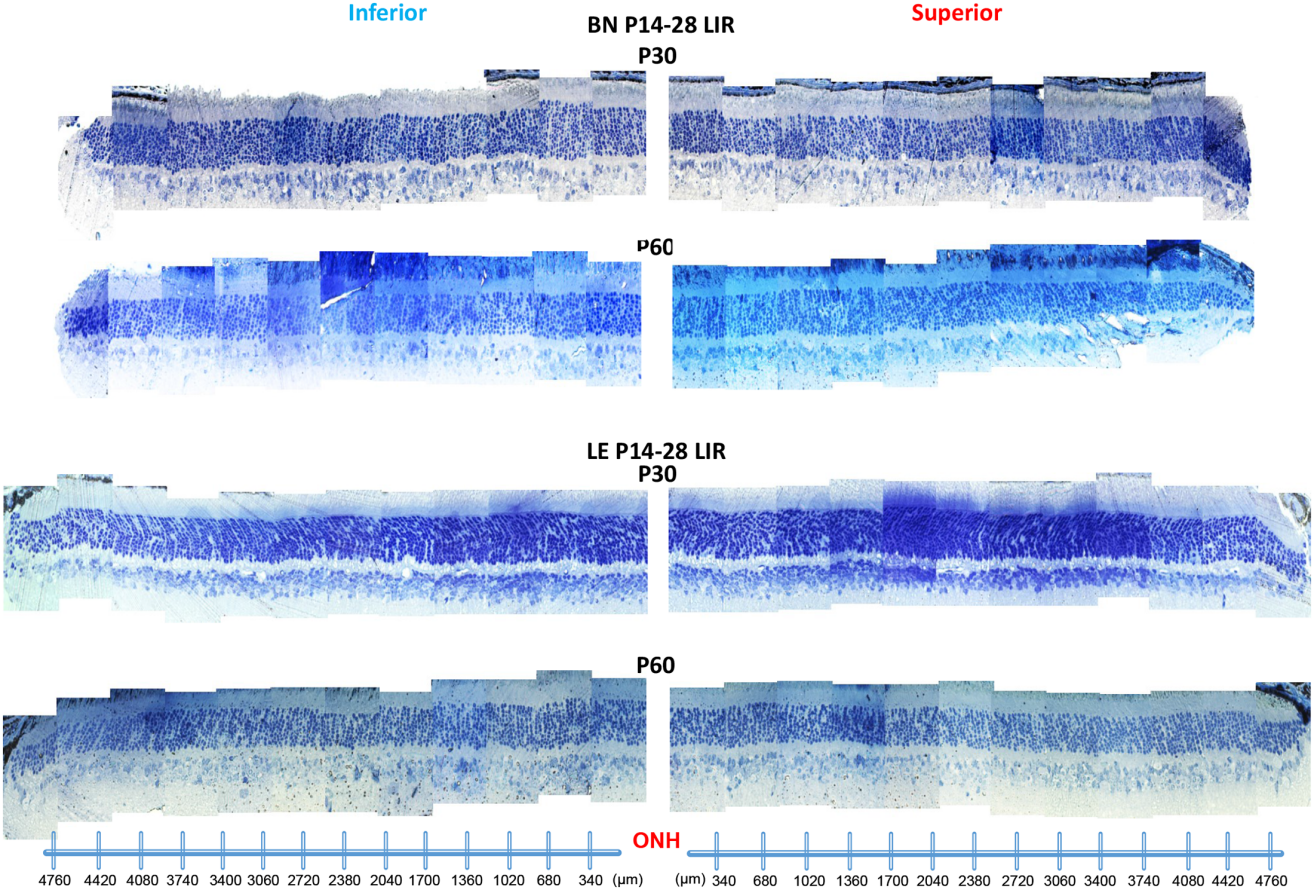
Representative reconstruction of the ONL of the inferior (left) and superior (right) retina (composed of 12–13 consecutive histological segments of 75μm in width, each sectioned at every 340μm from the ONH to the ora serrate of each hemiretina) obtained from control BN and LE rats at P30 and P60. Abbreviations: Optic nerve head (ONH), Brown Norway (BN), Long Evans (LE) and postnatal day (P).

**Fig 8:**
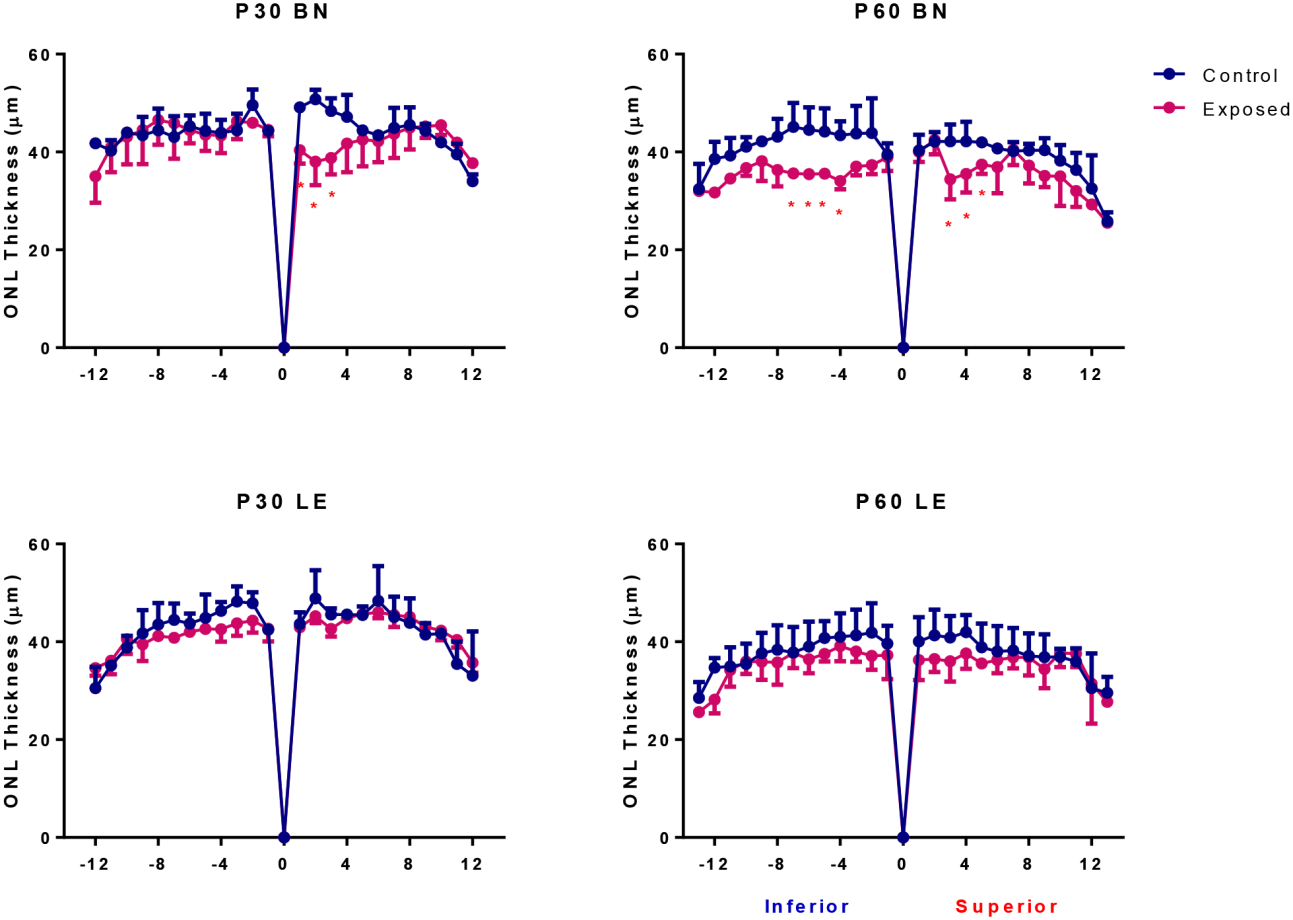
Spidergraph representation of ONL loss along the inferior (left) and superior (right) axis in control (blue) and light exposed (pink) BN and LE rats at P30 and P60. Measurements were taken at every 340μm from the optic nerve head towards the ora serrate in both hemiretinas. Abbreviations: Optic nerve head (ONH), Brown Norway (BN) and Long Evans (LE).

Fig 7 illustrate representative inferior (left) and superior (right) hemiretinas of control and light exposed BN and LE rats at age P30 and at P60. In control groups, the ONL thickness was relatively uniform across the entire vertical meridian examined and of similar (p>0.05) thickness in both strains irrespective of age (P30 and P60 measurements: Fig 8). The only age-related changes noted were those in the central retina (Segments 1, 2 and 5, 6; Fig 8) of BN rats, where the ONL layer was significantly thinner at P60 when compared to P30 values (p<0.05). Immediately following light exposure (at P30), no significant thinning of the ONL was observed in juvenile LE rats (Fig 8). In contrast, in juvenile BN rats, a small, but significantly thinner ONL (p<0.05) was observed in the superior retina between the ONH and 1020μm towards the ora serrata (Segments 1 to 3). This damage was however minimal to the one observed in adult BN rats. One month after the end of the light exposure (at P60), while no significant ONL damage could still be evidenced in LE rats, a gradual loss of photoreceptors occurred in BN rats (Fig 8). This ONL loss was now limited to the central retina of both the superior (between 680μm to 1700μm; segments 2 and 3) and the inferior (between 1360μm to 2400μm from the ONH; Segments 4 to 7) hemiretinas.

### Ocular pigmentation in juvenile and adult BN rats

**Fig 9:**
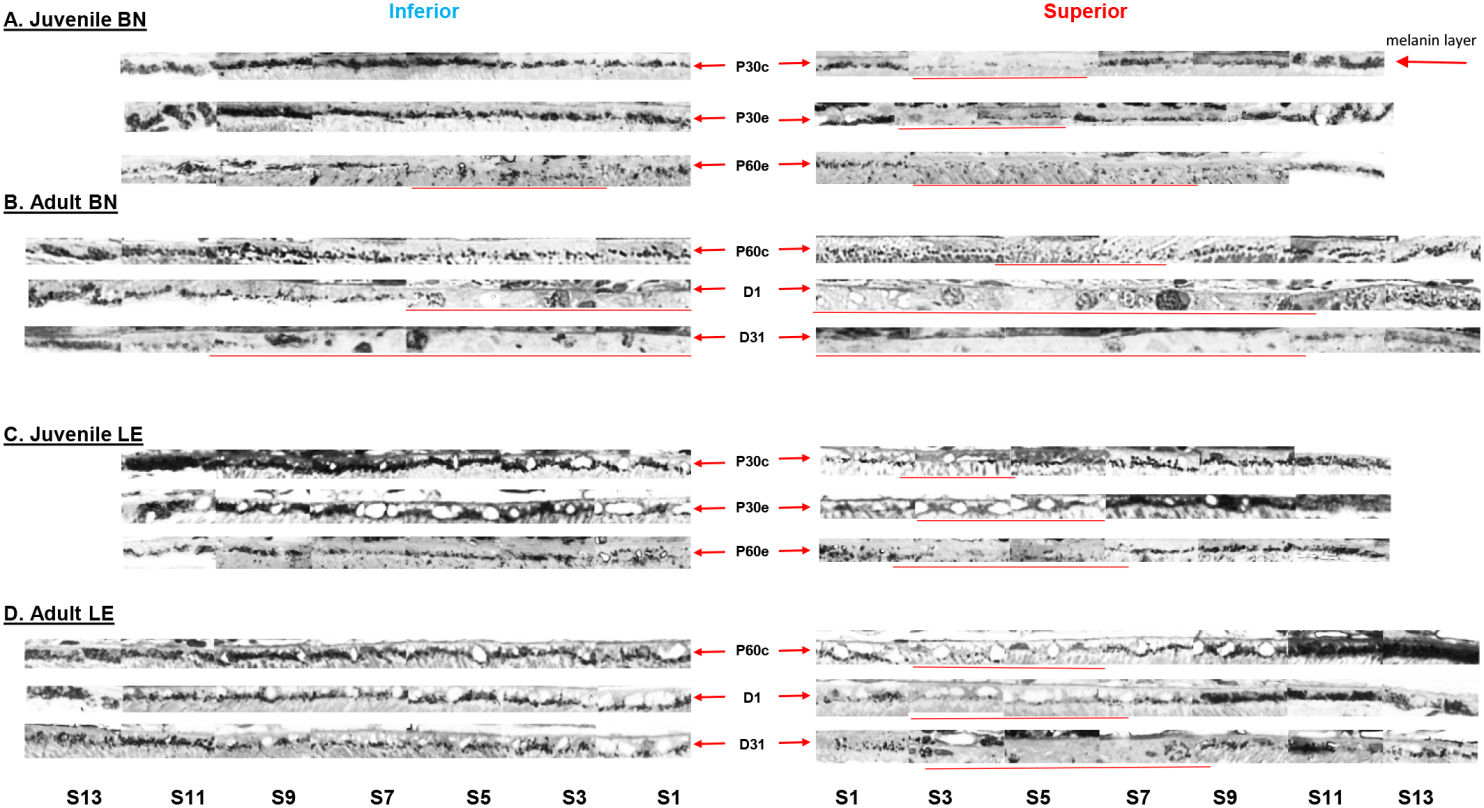
Representative histological samples of melanin pigment distribution along the inferior (left) and superior (right) hemiretinas in control and exposed juvenile (A) and adult (B) BN rats and juvenile (C) and adult (D) LE rats. Abbreviations: Segments (S), postnatal day (P), days after light exposure (D) and segment (S). Images were taken at every second segment. Each segment is 340μm in width. Red arrows identify the melanin pigment layer in both hemiretinas and red lines indicate the portion of the retina almost completely lacking the melanin pigment, as defined in the method section.

Fig 9 show representative histological sections showing melanin pigment distribution along the superior and inferior hemiretinas in control and exposed juvenile and adult BN and LE rats. The mean (average of all segments) melanin density of each hemiretina obtained using the melanin pigment scale is presented in Table 1.

**Table 1:** Mean (average of all segments) melanin density for each hemiretina (superior and inferior retinas) in control and exposed juvenile and adult rats. Abbreviations: Brown Norway (BN), Long Evans (LE), postnatal day (P) and days after the light exposure (D). Asterisks identify statistically significant differences (p<0.05) between exposed and control rats at age P60 for each hemiretina (as per one-way ANOVA analysis). Pound signs identify statistically significant differences between exposed adult BN and LE rats (as per one-way ANOVA analysis). Dollar signs identify statistically significant differences between the superior and inferior retinas of LE rats 31 days after the light exposure (as per t-test analysis).

	BN		LE	
	Superior	Inferior	Superior	Inferior
**Control at P30**	**2.79±0.06**	**3.25±0.23**	**2.71±0.25**	**3.46±0.15**
**Exposed at P30**	**2.60±0.21**	**3.23±0.04**	**2.42±0.05**	**3.26±0.04**
**Exposed at P60**	**2.22±0.40**	**2.82±0.46**	**2.43±0.38**	**3.13±0.12**
**Control at P60**	**2.56±0.25**	**3.21±0.19**	**2.42±0.24**	**3.35±0.18**
**Adult D1**	1.19±0.05*#	2.00±0.22*#	**2.49±0.34**	**3.32±0.11**
**Adult D31**	1.31±0.15*	1.79±0.24*#	1.85±0.57*$	**3.15±2.12**

In juvenile rats, irrespective of the age, the strain or the light exposure, no significant differences were observed when the total density of the melanin pigment (mean of all segments) was analyzed in each hemiretina, although the inferior retina tended to have a higher level of the melanin pigment compared to the superior retina (Table 1). Detailed analysis at each eccentricity revealed that, in both control and exposed BN and LE rats, a small portion of the superior retina was almost completely devoid of the melanin pigment. In control groups at P30, this melanin-free region extended between 680μm and 2040μm from the ONH [Segments 2 to 5] and was similar in both strains (Fig 9A and 9C, as indicated by the red lines). With time (at P60), while in LE rats this region remained relatively stable, in BN rats, an increase in size was noted (from 680μm to 2380μm; Segments 2 to 7) (Fig 9A and 9C). In contrast, the inferior retina presented with a relatively uniform distribution of the melanin pigment in all controls. In exposed BN and LE rats, a similar distribution of the melanin pigment was observed in both hemiretinas as in the control groups.

In contrast, the density of the melanin pigment was markedly more affected by the bright light exposure in adult rats (Fig 9B and 9D, as indicated by the red lines). In adult BN rats, a significant (p<0.05) loss of melanin pigment was already evidenced one day after the end of the light exposure (Table 1), where melanin pigments were only found at the far periphery of each hemiretina [region devoid of melanin pigments: from the ONH to 4120μm (superior; segments 1 to 12) and to 2140μm (inferior; segments 1 to 6; Fig 9B)]. Loss of melanin pigments (and consequently an increase in size of the melanin-free zone) was greater than the underlying photoreceptor-free zone in BN rats [region devoid of photoreceptors: from the ONH to 2020μm (inferior) and to 3700μm (superior), Fig 5]. With time (D31), an increase in size of the melanin-free zone occurred only in the inferior retina (from the ONH to 2720μm; segments 1 to 8; Fig 9B).

In adult LE rats, one day after the light exposure, no significant differences in melanin pigment density were noted with control groups (Table 1 and Fig 9D). However, one month after the light exposure (at D31), lower levels of melanin pigment were found in the superior retina compared to control (p>0.05). As in adult BN rats, an increase of the melanin-free zone was noted, but was limited to the superior retina only [region devoid of melanin pigments in LE rats (superior retina only): D1: 680μm (from the ONH) to 1935μm; segments 2 to 6 and D30: from 290μm to 2700μm; segments 1 to 8]. The distribution of the melanin pigment in the inferior retina remained relatively uniform with age and within control values (Fig 9D and Table 1).

## Discussion

In the present study, we examined the role of ocular pigmentation and genetics in light-induced retinal damage by comparing inbred (Brown Norway and Lewis rats) and outbred (Sprague-Dawley and Long-Evans) albino (Lewis and Sprague-Dawley) and pigmented (Brown Norway and Long-Evans) rat stains. Our results show that of all the strain tested, adult BN rats were the most susceptible to photooxidative stress. Previous studies have also reported the high susceptibility of the adult BN retina to light damage [5,15]. However, our findings also revealed that when exposed at a younger age, BN rats were almost as resistant to light damage as juvenile LE rats, suggesting that this susceptibility to bright light develops as the BN rat ages. Our results thus strongly suggest that, although ocular pigmentation and genetic background are important factors that regulate the severity of light-induced retinal damage, the age of the animal at the onset of exposure appears to be a more important determining factor.

### Comparing the susceptibility to light damage in four different strains of rats

Ocular pigmentation in adult BN rats was not as efficient in protecting the retina against the bright light insult as it did for LE rats. Interestingly, although the rod and cone function was severely affected by the light exposure in all four strains, in BN rats, cone function was more affected than rod function. Furthermore, our follow-up studies revealed a recovery only for the rod function, a finding true for both the adult and the juvenile BN rats. This contrasts with the other strains, where a recovery was observed for both photoreceptors. Although it was shown that rod outer segments are the primary target of light-induced oxidative stress [16] and that cones are the photoreceptors most resistant in this retinopathy [17], strain dependent variations in the intrinsic properties of the photoreceptors might explain these differences. For example, histological data revealed that BN rats had longer inner segments compared to the other three strains (S3 Fig). Since the inner segment is involved in the formation of new outer segments discs (where the visual pigment is stored) as well as in the synthesis of visual pigment molecules [18–19], it could be that the retina of BN rats generates higher amounts of rhodopsin and consequently is equipped with a better capacity to catch photons. This would be supported by larger scotopic a-wave amplitudes recorded in BN rats compared to other strains. Since rhodopsin can act as a chromophore during light exposure [20], the degree of light damage is therefore dependent upon the availability of functional rhodopsin: more visual pigment equals more damage. Consequently, the longer inner segments of BN rats might predispose their retina to a more severe light-induced damage. This hypothesis would also be supported by the higher amounts of the RPE65 protein measured in BN rats compared to WS, LW and LE rats [21]. The RPE65 protein is an important component of the visual cycle that is implicated in the regeneration of rhodopsin [22–23]. In mice, the availability of RPE65 was shown to modulate retinal susceptibility to light damage [24]; higher concentrations causing a more severe retinopathy (due to a faster regeneration and, thus, increased availability of rhodopsin). However, the level of RPE65 in albino rats did not correlate with the degree of retinal damage reported by Iseli et al. [21], where more damage was observed in WS rats which has a lower level of RPE65 protein compared to LW rats. They concluded that at least in rats, the RPE65 protein was not a significant factor in modulating the severity of LIR. This contrasts with the findings of Borges et al., 1990 [25] who showed a higher resistance to light damage in WS compared LW rats, findings that would agree with the difference in RPE65 protein expression reported by Iseli and his group. Furthermore, given that Iseli et al. did not evaluate the relationship between RPE65 and light susceptibility in the pigmented strains, we cannot exclude the possibility that in other strains such as in BN rats, higher levels of this protein might lead to a greater retinal damage. Previously reported differences in the gene sequence of the RPE65 protein, such as the substitution of the leucine by methionine amino acid at position 450, might propose another explanation for these stain differences [24,26]. Thus, based on the above, the higher susceptibility of BN rats’ retina to light damage might reflect some intrinsic differences at the level of the visual cycle, such as differences in the availability or variations in the gene sequence of the RPE65 protein. In contrast, the severity of light damage was less different between the two albinos strains; photoreceptor loss being slightly greater in SD compared to LW rats. To our knowledge, no study has compared the level of the RPE65 protein in these two strains.

VEGF (vascular endothelial growth factor), a growth factor implicated in the angiogenesis, was also reported to be higher in BN rats, compared to other pigmented (LE rats) as well as albino (SD and WS rats) strains. The latter difference is believed to be the reason behind the more significant vascular abnormalities (i.e. larger avascular region, greater vascular tortuosity and constriction, higher incidence of neovascularization and vascular leakage) observed in this strain [27–29]. A recent study by Cachafeiro et al., 2013 [30] showed that following a bright light insult, the activation of VEGF signalling leads to the breakdown of the outer blood-retinal barrier (and an increase in the RPE hyperpermeability) and photoreceptor apoptosis. Thus, differences in VEGF expression between strains might propose another mechanism at the origin of these strain-related retinal variations and might explain why a higher retinal susceptibility is observed in adult BN rats. In contrast, in juvenile BN rats, due to their higher resistance to light damage, the intensity of light or the resulting oxidative stress might be not sufficient to activate this pathway and thus render the younger retinas less prone to light damage.

### Comparing the susceptibility to light damage of juvenile and adult BN rats

We demonstrated on several occasions that young animals are more resistant to light-induced damage compared to older animals [6,12], findings obtained using SD strains. In the present study, we wished to determine if juvenile BN rats also showed a similar resistance. Furthermore, as adult BN rats showed more damage compared to albino rats, we also wanted to know whether this strain was more susceptible to light damage form birth or whether this higher vulnerability was a feature that these animals acquired as they aged.

Although photoreceptor damage in adult BN rats was the most severe among the different strains tested, juvenile BN rats were almost as resistant to light damage as juvenile LE rats. Even one month after the end of light exposure, although a greater damage was observed in juvenile BN rats compared to juvenile LE rats, photoreceptor loss was still less pronounced compared to that found in the albino strains. These findings suggest that the higher susceptibility to retinal light damage in BN rats is a feature that these animals acquire with age. Although in control groups, ONL thickness was similar in both strains and no age-related changes were observed between P30 and P60 (Fig 8), it could still be that with age the susceptibility of photoreceptors is enhanced due to genetic factors or that the accumulated oxidative stress changes the biochemical properties of the melanin pigment of BN rats.

In addition to the age factor, the intensity of the light exposure also appears to play an important factor in strain-related retinal susceptibility to light damage. In the present study, as well as in the studies by Tremblay et al., 2013 and Jamison and Vihtelic, 2011 [5,15], when adult BN rats were compared with albino rats, the light intensity was always equal or above 10,000lux [10,000lux, 18,000lux and 19,000lux, respectively] and the damage was always greater in adult BN rats. However, when Humpel et al., 1992 [31] exposed adult BN rats to 2,500 and 6,100lux for 21 days, the retina of BN rats was intact, while that of SD rats showed a severe photoreceptor destruction. The major difference in their study was the illumination level, which was much lower. It could be that at a lower light intensity, less oxidative stress is generated in BN retinas, levels that would be similar to those observed in juvenile BN rats, and consequently explain why in Humpel et al.’s study the adult BN rats are as resistant to light damage as are the juvenile BN rats in the present study. This in turn could suggest that a minimal threshold of oxidative damage must be reached in order to induce severe damage to the adult BN retina. Thus, under higher light levels, the melanin pigment could lose its anti-oxidant properties and become pro-oxidant (more free radicals generated) and consequently, predispose the adult BN retinas to a greater photoreceptor loss. Together, these findings suggest that both the age and the intensity of the light exposure greatly influence the degree of retinal damage in this strain.

### Melanin protection in BN and LE rats

In both adult BN and LE rats, a similar portion of the superior retina is devoid of melanin pigments, a region that we termed the “melanin free zone” (between 680μm and 2380μm and between 680μm and 2040μm from the ONH, respectively), suggesting that, initially, both retinas would be equally vulnerable to light damage. However, compared to adult LE rats, most of the melanin pigments of BN retinas were destroyed by the light exposure (only few pigments remained at the periphery). This contrast with juvenile BN and LE rats, where a similar density of the melanin pigment was observed following light exposure. Thus, a greater loss of the melanin pigment as observed in adult BN rats, might predispose these photoreceptors to a higher photon absorption (since less protection and light absorption is provided by the melanin pigment) and consequently lead to a more severe light-induced damage.

The higher vulnerability of the retina of BN rats might also be due to their type of melanin. In vertebrate retina, two types of melanin pigments are found at the level of the RPE: eumelanin and pheomelanin [32–33]. Previous studies showed that eumelanin possess better photoprotective properties compared to pheomelanin [34–36]. To our knowledge, so far no study has evaluated the distribution of these two pigments in BN and LE rats. It could be that the RPE of BN rats contains more pheomelanin than eumelanin and consequently, the BN retina is prone to a higher oxidative stress.

As shown above, melanin pigmentation does not successfully protect the retina in all pigmented strains, suggesting that some strains, including albino ones, might benefit from a more favorable genetic background to fight against the light-induced oxidative stress. As mentioned above, the BN rat is an inbred strain, meaning that they have almost an identical genotype (a consecutive 20 long generation mating produce a homozygosity of approximatively 98%). Consequently, these animals are more predisposed to transmit mutated recessive genes to their offsprings and thus significantly increase the risk to develop different anomalies, including transmissible retinopathies. In contrast, the pigmented LE rat is an outbred strain (breeding two animals distantly related or unrelated) and consequently the risk of such transmission is reduced (increase of heterozygosity), thus providing this strain with a natural resistance. Furthermore, while LW, SD and LE rats appear to have the Wistar rat as a common ancestor, the BN rat does not. Since the Wistar rat is an outbred strain, while the BN is an inbred strain, this could also explain why the BN rat is more vulnerable (information taken from the Rat Genome Database website: http://rgd.mcw.edu/). However, although both ocular pigmentation and genetics significantly modulate the degree of retinal damage, the age at the onset of the retinopathy also play a crucial role in that retinal susceptibility to light damage. The importance of the age factor as a modulator of retinal damage is also a finding that agrees with results obtained from other studies on animal models of oxidative stress, such as the oxygen-induced retinopathy [37].

### Strain-related long term consequences of bright light exposure

Our long term data shows that LE rats were most resistant to light damage, irrespective of the age. Even after more than a year following the cessation of bright light exposure, the retina of LE rats still presented with a relatively well preserved function and structure. Interestingly, an accumulation of some inner retinal cells was evidenced. These cells are most probably either remaining amacrine or bipolar cells, as ganglion cells were shown to degenerate within 6 months post-light exposure [38]. Loss of RGC was attributed to displaced retinal vessels that invaded the retina at the optic nerve head and compressed the axons of RGC thus causing their death. However immunohistological staining will be needed for a precise identification of these cells.

In LW rats, no outer retina remained after one year post-light exposure. In addition, the remaining inner retina was significantly disorganized. Traces of blood vessel invasion, retinal scaring and vacuolization were some of the features observed in the remaining inner retina (Fig 4). Furthermore, most of the cells of the inner retina were distributed randomly in the remaining retina. Interestingly, although the sampled retinal regions of LW rats completely lacked of an outer retina 1-year post exposure, an a-wave of 10% of normal amplitude could still be recorded (Fig 2), suggesting the possibility that some photoreceptors may have survived in other (un-sampled) regions of the retina, accounting for approximately 10% of normal retinal surface.

A quantitative summary of all the above mentioned findings can be found in S2 Table.

## Conclusion

Regardless of the degree of pigmentation, light exposure significantly impaired the retinal function and structure in all four strains. However, when light exposure took place at an adult age, the pigmented LE rats were the most resistant to light damage and the pigmented BN rats were the most severely damaged. A different pattern occurred when rats were exposed at a younger age, where BN rats were almost as resistant as LE rats. Our results thus reveal that the enhanced susceptibility of photoreceptors to light damage seen in adult BN rats is acquired as the rat ages. Thus, the age of the animal at the onset of the light exposure should be considered when comparing the degree of retinal damage between different strains. Furthermore, although ocular pigmentation might be efficient in protecting the retina in some strains, other factors such as strain differences in intrinsic properties of the photoreceptors, other biomolecular factors and genetics must be taken in consideration in order to explain the above differences.

## Supporting Information

S1 Fig Enlarged view of selected histological sections of the inferior (left) and superior (right) retina (taken at 1000μm directly above and below the ONH) obtained from four different strains of adult light-exposed rats 1 day and 31 days following light exposure. Abbreviations: Outer nuclear layer (ONL), inner nuclear layer (INL), Brown Norway (BN), Sprague-Dawley (SD), Lewis (LW) and Long Evans (LE). Calibration bar: 75μm.
(TIF)

S2 Fig Enlarged view of selected histological sections from the superior and the inferior retinas (taken at approximatively 300, 1700 and 4700μm from the ONH) 1 year post-light exposure in LW and LE rats. All sections are aligned with the Bruch’s membrane (blue arrow). In LW rats, the retina is completely devoid of photoreceptors and is significantly disorganized (inner retinal and choroidal vessels invasion, retinal scaring and vacuolization). Specific retinal layers are no longer distinguishable in LW rats. Damage in LE rats is less severe and photoreceptors are still noticeable at the far periphery in both hemiretinas. Abbreviations: Bruch’s membrane (BRM), retinal pigment epithelium (RPE), outer segment (OS), inner segment (IS), outer nuclear layer (ONL), outer plexiform layer (OPL), inner nuclear layer (INL), inner plexiform layer (IPL), retinal ganglion cell and fiber layer (RGC/FL), Lewis (LW) and Long Evans (LE). Calibration bar: 75μm.
(TIF)

S3 Fig Representative sections of the superior retinas (taken at 1000μm directly above and below the ONH) taken from control rats of each strain. Abbreviations: Optic nerve head (ONH), Brown Norway (BN), Lewis (LW), Sprague-Dawley (SD) and Long Evans (LE). Asterisks illustrate statistically significant differences in the inner segment length (p<0.05) between BN rats and other strains. Calibration bar: 75μm.
(TIF)

S1 Table Intensity of RodVmax following bright light exposure in BN, SD, LW and LE rats. Intensity in log.cd.m^-2^. No rodVmax could be measured in D31 BN rats. Only LW and LE rats were tested at long term. Abbreviations: Brown Norway (BN), Sprague-Dawley (SD), Lewis (LW), Long Evans (LE) and days after the light exposure (D). Asterisks identify statistically significant differences (p<0.05) between exposed and control rats of respective groups at D1 and D31. Dollar signs identify statistically significant differences between exposed adult LE rats and the other three stains at D1. Pound signs identify statistically significant differences between exposed adult LE rats and the other three stains at D31 (as per one-way ANOVA analysis).
(DOCX)

S2 Table Quantitative summary of all structural and functional changes observed in four different strains of rats following bright light exposure. Abbreviations: Days post-light exposure (D), Inner segment (IS), Brown Norway (BN), Sprague-Dawley (SD), Lewis (LW), Long Evans (LE) and not applicable (N/A).
(DOCX)

## References

[1] Noell WK, Walker VS, Kang BS, Berman S. Retinal damage by light in rats. *Invest*. *Ophthalmol*. *Vis Sci*. 1966;5:450–473. 5929286

[2] Lavail MM. Eye pigmentation and constant light damage in the rat retina Williams et al (eds), *The effects of constant light on visual processes*. Plenum Press, New York 1980;357–387.

[3] Wascowicz M, Morice C, Ferrari P, Callebert J, Versaux-Botteri C. Long-term effects of light damage on the retina of albino and pigmented rats. *Invest Ophthalmol Vis Sci*. 2002;43:813–820. 11867603

[4] LaVail MM, Gorrin GM, Repaci MA, Thomas LA, Ginsberg HM. Genetic regulation of light damage to photoreceptors. *Invest*. *Ophthalmol*. *Vis*. *Sci*. 1987; 7;28(7):1043–8. 3596986

[5] Tremblay F, Waterhouse J, Nason J, Kalt W. Prophylactic neuroprotection by blueberry-enriched diet in a rat model of light-induced retinopathy. *Journal of nutritional biochemistry*. 2013;24:647–655. 10.1016/j.jnutbio.2012.03.011 22832077

[6] Joly S, Pernet V, Dorfman AL, Chemtob S, Lachapelle P. Light-induced retinopathy: comparing adult and juvenile rats. *Invest Ophthalmol Vis Sci*. 2006;47:3202–3212. 16799068 10.1167/iovs.05-1515

[7] O'Steen WK, Anderson KV, Shear CR. Photoreceptor degeneration in albino rats: dependency on age. *Invest Ophthalmol Vis Sci*. 1974;13(5):334–339. 4823176

[8] Kuwabara T, Funahashi M. Light damage in the developing rat retina. *Arch Ophthalmol*. 1976;94(8):1369–1374. 949280 10.1001/archopht.1976.03910040237017

[9] Malik S, Cohen D, Meyer E, Perlman I. Light damage in the developing retina of the albino rat: an electroretinographic study. *Invest Ophthalmol Vis Sci*. 1986;27(2):164–167. 3943942

[10] Rapp LM and Williams TP. The role of ocular pigmentation in protecting against retinal light damage. *Vision Research*. 1980;20:1127–1131. 7269270 10.1016/0042-6989(80)90050-4

[11] Penn JS, Anderson PE. Effect of light history on rod outer segment membrane composition in the rat. *Exp Eye Res*. 1987;44:767–778. 3653272 10.1016/s0014-4835(87)80040-4

[12] Polosa A, Liu W, Lachapelle P. Retinotopic distribution of structural and functional damages following bright light exposure of juvenile rats. PLos One. 2016;11(1): 1–20. 10.1371/journal.pone.0146979 PMC4718541 26784954

[13] Williams RA, Howard AG, Williams TP. Retinal damage in pigmented and albino rats exposed to low levels of cyclic light following a single mydriatic treatment. *Curr Eye Res*. 1985;4(2):97–102. 3987351 10.3109/02713688508999974

[14] Hébert M, Lachapelle P, Dumont M. Reproducibility of electroretinograms recorded with DTL electrodes. *Doc*. *Ophthalmol*. 1996;91:333–342. 10.1007/BF01214651 8899303

[15] Jamison JA, Vihtelic TS. Blue light damage susceptibility of albino and pigmented rats strains. *Invest Ophthalmol Vis Sci*. 2011;52: E-abstract: 1849

[16] Remé CE. The dark side of light: rhodopsin and the silent death of vision. *Invest*. *Ophthalmol*. *Vis*. *Sci*. 2005;46:2672–2682. 10.1167/iovs.04-1095 16043837

[17] Cicerone CM. Cones survive rods in the light-damaged eye of the albino rat. Science. 1976;194(4270):1183–1185. 996550 10.1126/science.996550

[18] Matsubara T, Miyata M, Mizuno K. Radioisotopic studies on renewal of opsin. Vision Res. 1968;8(9):1139–1143. 5682791 10.1016/0042-6989(68)90023-0

[19] Hall MO, Bok D, Bacharach DE. Biosynthesis and assembly of the rod outer segment membrane system. Formation and fate of visual pigment in the frog retina. J Mol Biol. 1969;45:397–406. 5367035 10.1016/0022-2836(69)90114-4

[20] Grimm C, Wenzel A, Hafezi F, Yu S, Redmond TM, Remé CE. Protection of Rpe65-deficient mice identifies rhodopsin as a mediator of light-induced retinal degeneration. Nat Genet. 2000;25(1):63–66. 10802658 10.1038/75614

[21] Iseli H-P, Wenzel A, Hafezi F, Reme CE, Grimm C. Light damage susceptibility and RPE65 in rats. *Exp*. *Eye Res*. 2002;75:407–413. 12387788

[22] Redmond TM, Yu S, Lee E, Bok D, Hamasaki D, Chen N, et al RPE65 is necessary for production of 11-cis-vitamin A in the retinal visual cycle. *Nat*. *Genet*. 1998;20:344–351. 9843205 10.1038/3813

[23] Moiseyev G, Chen Y, Takahashi Y, Wu BX, Ma J-X. RPE65 is the isomerohydrolase in the retinoid visual cycle. *Proc*. *Natl*. *Acad*. *Sci*. 2005;102:12413–12418. 16116091 10.1073/pnas.0503460102 PMC1194921

[24] Wenzel A, Remé CE, Williams TP, Hafezi F, Grimm C. The RPE65 Leu450Met variation increases retinal resistance against light-induced degeneration by slowing rhodopsin regeneration. *The Journal of Neuroscience*. 2001;21(1):53–58. 11150319 10.1523/JNEUROSCI.21-01-00053.2001 PMC6762429

[25] Borges JM, Edward DP, Tso MO. A comparison study of photic injury in four inbred strains of albino rats. Curr Eye Res. 1990;9(8):799–803. 2276280 10.3109/02713689008999576

[26] Danciger M, Matthes MT, Yasumura D, Akhmedov NB, Rickabaugh T, Gentleman S et al, A QTL on distal Chr 3 that influences the severity of light-induced damage to mouse photoreceptors. *Mamm*. *Genome*. 2000;11:422–427. 10818205 10.1007/s003350010081

[27] Gao G, Li Y, Fant J, Crosson CE, Becerra SP, Ma JX. Difference in ischemic regulation of vascular endothelial growth factor and pigment epithelium—derived factor in Brown Norway and Sprague Dawley rats contributing to different susceptibilities to retinal neovascularization. *Diabetes*. 2002; 51:1218–25. 11916948 10.2337/diabetes.51.4.1218

[28] Floyd BNI, Leske DA, Wren SME, Mookadam M, Fautsch MP, Holmes JM. Differences between rat strains in models of retinopathy of prematurity. *Molecular Vision*. 2005;11:524–530. 16052168

[29] Zhang SX, Ma JX, Sima J, Chen Y, Hu MS, Ottlecz A, et al Genetic difference in susceptibility to the blood-retina barrier breakdown in diabetes and oxygen-induced retinopathy. *American Journal of Pathology*. 2005;166: 313–321. 15632023 10.1016/S0002-9440(10)62255-9 PMC1602304

[30] Cachafeiro M, Bemelmans A-P, Samardzija M, Afanasieva T, Pournaras J-A, Grimm C, et al Hyperactivation of retina by light in mice leads to photoreceptor cell death mediated by VEGF and retinal pigment epithelium permeability. *Cell death and Disease*. 2013; 4: e781:1–11. 10.1038/cddis.2013.303 23990021 PMC3763463

[31] Humpel C, Neudorfer C, Philipp W, Steiner HJ, Haring C, Schmid KW, et al Effects of bright artificial light on monoamines and neuropeptides in eight different brain regions compated in a pigmented and nonpigmented rat strain. Journal of Neuroscience Research. 1992;32:605–612. 1527805 10.1002/jnr.490320416

[32] Wielgus AR, Sarna T. Melanin in human irides of different color and age of donors. *Pigment Cell Res*. 2005;18:454–464. 16280011 10.1111/j.1600-0749.2005.00268.x

[33] Ito S, Pilat A, Gerwat W, Skumatz CMB, Ito M, Kiyono A, et al Photoaging of human retinal pigment epithelium is accompanied by oxidative modification of its eumelanin. *Pigment Cell Melanoma Res*. 2013;26:357–366. 10.1111/pcmr.12078 23421783

[34] Prota G. The chemistry of melanins and melanogenesis. Barrera JB et al, *Fortschritte der Chemie organischer Naturstroffe/Progress in the chemistry of organic natural products*. 1995;93–148. 10.1007/978-3-7091-9337-2_2 7782013

[35] Brenner M, Hearing VJ. The protective role of melanin against UV damage in human skin. *Photochemistry and Photobiology*. 2008;84:539–549. 10.1111/j.1751-1097.2007.00226.x 18435612 PMC2671032

[36] Simon JD, Peles DN. The red and the black. *Accounts of chemical research*. 2010;43(11):1452–1460. 10.1021/ar100079y 20734991

[37] Dembinska O, Rojas LM, Chemtob S, Lachapelle P. Evidence for a brief period of enhanced oxygen susceptibility in the rat model of oxygen-induced retinopathy. Invest Ophthalmol. Vis Sci. 2002;43:2481–2490. 12091454

[38] Garcίa-Ayuso D, Salinas-Navarro M, Agudo-Barriuso M, Alarcόn-Martinez L, Vidal-Sanz M, Villegas-Pérez MP. Retinal ganglion cell axonal compression by retinal vessels in light-induced retinal degeneration. Mol Visc. 2011;17:1716–1733. PMC3130728 21738401

