# Supplementary figures and images for: Strain Differences in Light-Induced Retinopathy
Source: PLoS One. 2016 Jun 29;11(6):e0158082. doi: 10.1371/journal.pone.0158082 (PMC4927188; doi:10.1371/journal.pone.0158082)

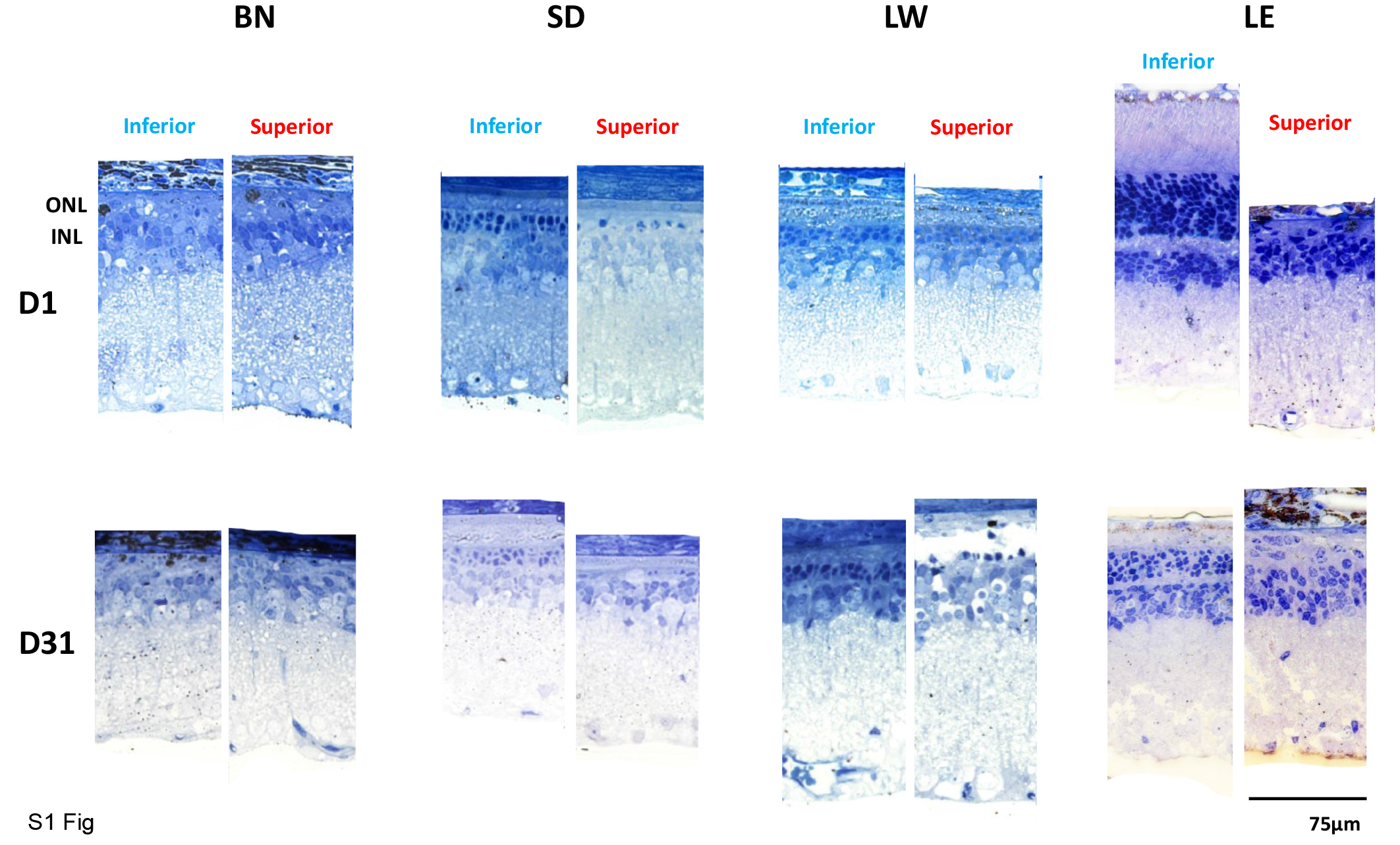

Supplement: S1 Fig — Abbreviations: Outer nuclear layer (ONL), inner nuclear layer (INL), Brown Norway (BN), Sprague-Dawley (SD), Lewis (LW) and Long Evans (LE). Calibration bar: 75μm. (TIF) [file pone.0158082.s001.tif]

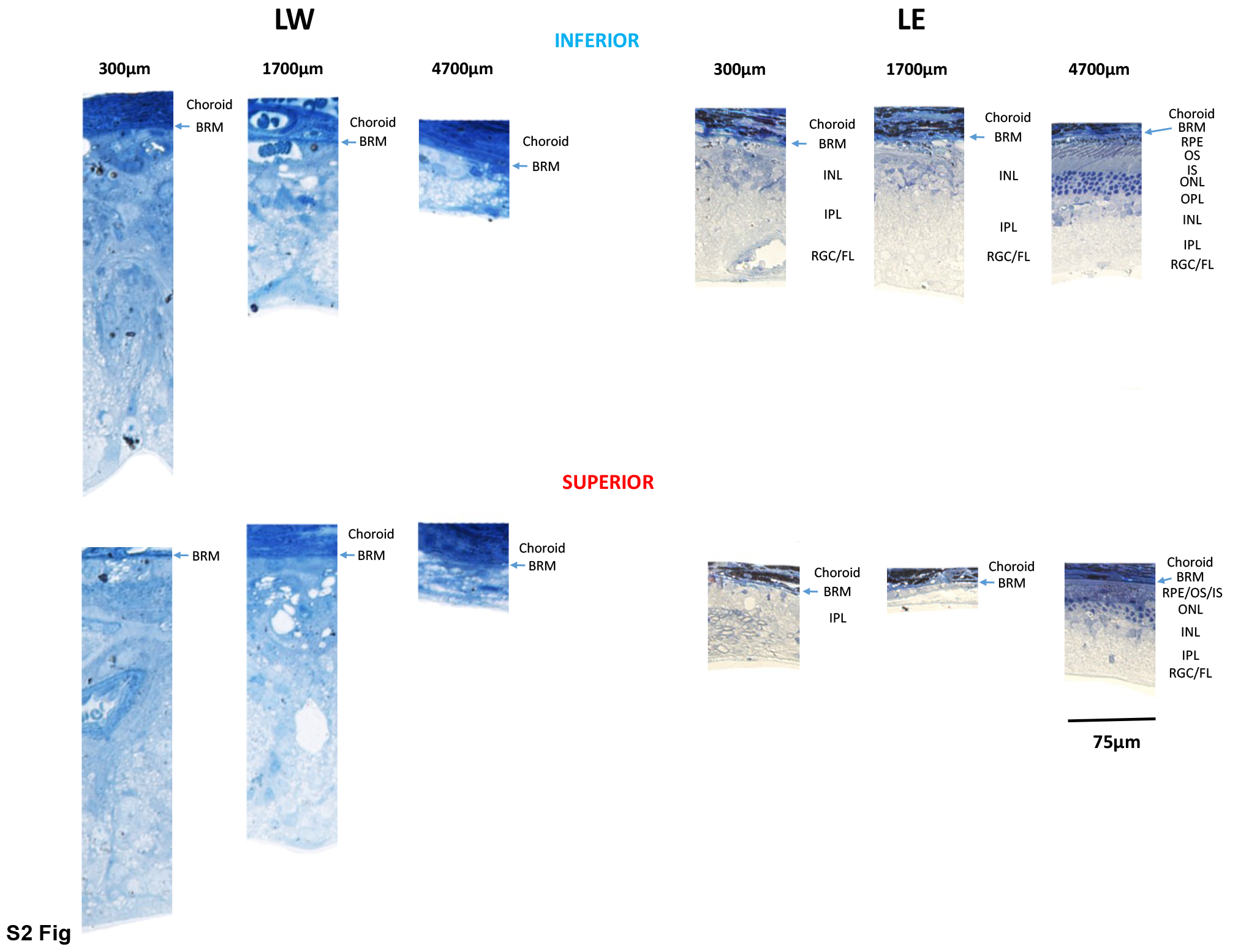

Supplement: S2 Fig — All sections are aligned with the Bruch’s membrane (blue arrow). In LW rats, the retina is completely devoid of photoreceptors and is significantly disorganized (inner retinal and choroidal vessels invasion, retinal scaring and vacuolization). Specific retinal layers are no longer distinguishable in LW rats. Damage in LE rats is less severe and photoreceptors are still noticeable at the far periphery in both hemiretinas. Abbreviations: Bruch’s membrane (BRM), retinal pigment epithelium (RPE), outer segment (OS), inner segment (IS), outer nuclear layer (ONL), outer plexiform layer (OPL), inner nuclear layer (INL), inner plexiform layer (IPL), retinal ganglion cell and fiber layer (RGC/FL), Lewis (LW) and Long Evans (LE). Calibration bar: 75μm. (TIF) [file pone.0158082.s002.tif]

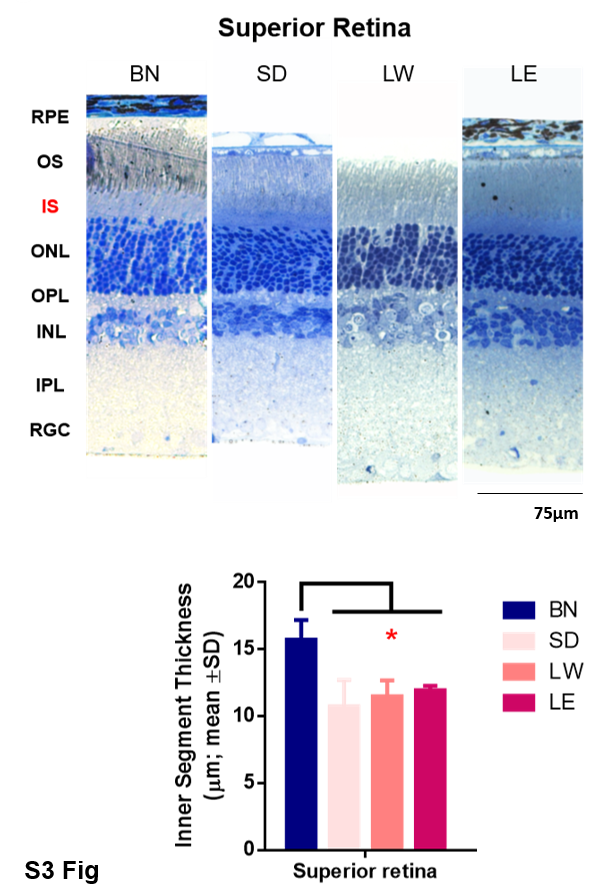

Supplement: S3 Fig — Abbreviations: Optic nerve head (ONH), Brown Norway (BN), Lewis (LW), Sprague-Dawley (SD) and Long Evans (LE). Asterisks illustrate statistically significant differences in the inner segment length (p<0.05) between BN rats and other strains. Calibration bar: 75μm. (TIF) [file pone.0158082.s003.tif]
