# Supplementary material for: Strain Differences in Light-Induced Retinopathy
Source: PLoS One. 2016 Jun 29;11(6):e0158082. doi: 10.1371/journal.pone.0158082 (PMC4927188; doi:10.1371/journal.pone.0158082)
Supplement: S1 Table — Intensity in log.cd.m-2. No rodVmax could be measured in D31 BN rats. Only LW and LE rats were tested at long term. Abbreviations: Brown Norway (BN), Sprague-Dawley (SD), Lewis (LW), Long Evans (LE) and days after the light exposure (D). Asterisks identify statistically significant differences (p<0.05) between exposed and control rats of respective groups at D1 and D31. Dollar signs identify statistically significant differences between exposed adult LE rats and the other three stains at D1. Pound signs identify statistically significant differences between exposed adult LE rats and the other three stains at D31 (as per one-way ANOVA analysis). (DOCX) [file pone.0158082.s004.docx]

| **(log.cd.m-^2^)** | **BN** | **SD** | **LW** | **LE** |
| --- | --- | --- | --- | --- |
| **Control** | -2.1±0.6 | -2.2±0.5 | -2.5±0.2 | -2.1±0.5 |
| **D1** | 0.9±0.0*$ | 0.9±0.0*$ | 0.9±0.0* | -1.1±0.3 |
| **D31** | non recordable | 0.9±0.0*# | -0.2±0.2* | -1.2±0.4 |
| **Long Term** | not performed | not performed | -0.4±1.1* | -0.9±0.0 |

S1 Table
