# Supplementary material for: Strain Differences in Light-Induced Retinopathy
Source: PLoS One. 2016 Jun 29;11(6):e0158082. doi: 10.1371/journal.pone.0158082 (PMC4927188; doi:10.1371/journal.pone.0158082)
Supplement: S2 Table — Abbreviations: Days post-light exposure (D), Inner segment (IS), Brown Norway (BN), Sprague-Dawley (SD), Lewis (LW), Long Evans (LE) and not applicable (N/A). (DOCX) [file pone.0158082.s005.docx]

|  | **Adult BN** | **Adult SD** | **Adult LW** | **Adult LE** |
| --- | --- | --- | --- | --- |
| **Retinal function**  **D1** | residual ERG | residual ERG | residual scotopic ERG and  reduced photopic ERG  (similar to LE) | residual scotopic ERG and reduced photopic ERG  (similar to LW) |
| **Retinal function**  **D31** | residual ERG | Partial recovery | Partial recovery  (more than SD) | Partial recovery  (similar to LW) |
| **SUPERIOR RETINA**  **(Photoreceptor layer)**  **D1** | Almost completely devoid of photoreceptors, except far periphery | Single row | 2 to 4 nuclei thick ONL | Only a small area is devoid  of photoreceptors centrally |
| **INFERIOR RETINA**  **(Photoreceptor layer)**  **D1** | Almost completely devoid of photoreceptors, except far periphery | 2 to 4 nuclei thick ONL | 2 to 4 nuclei thick ONL | No significant ONL thinning |
| **SUPERIOR RETINA**  **(Photoreceptor layer)**  **D31** | Similar as at D1  Longest IS | Almost completely devoid of photoreceptors | Only a small area is devoid of photoreceptors centrally | Similar damage as found in LW rats at D31 |
| **INFERIOR RETINA**  **(Photoreceptor layer)**  **D31** | Similar as at D1  Longest IS | 2 to 4 nuclei thick ONL | 2 to 4 nuclei thick ONL | 4 to 12 nuclei thick ONL |
| **RETINAL HISTOLOGY**  **Long Term data** | not performed | not performed | Completely devoid of photoreceptors; significant disorganization of remaining retina | Most of the superior retina and the central part of the inferior retina destroyed |
| **Melanin Pigmentation**  **D1** | Almost completely devoid of melanin pigments, except far periphery | N/A | N/A | No significant change  from control group |
| **Melanin Pigmentation**  **D31** | Same as at D1 | N/A | N/A | Increase of the region naturally lacking  the melanin pigment |
